# Comprehensive DNA methylation analysis of the *Aedes aegypti* genome

**DOI:** 10.1038/srep36444

**Published:** 2016-11-02

**Authors:** Cassandra Falckenhayn, Vitor Coutinho Carneiro, Anderson de Mendonça Amarante, Katharina Schmid, Katharina Hanna, Seokyoung Kang, Mark Helm, George Dimopoulos, Marcelo Rosado Fantappié, Frank Lyko

**Affiliations:** 1 Division of Epigenetics, DKFZ-ZMBH Alliance, German Cancer Research Center, 69120 Heidelberg, Germany; 2 Instituto de Bioquímica Médica Leopoldo de Meis, Universidade Federal do Rio de Janeiro, Rio de Janeiro, 21941-902, Brazil; 3 Institute of Pharmacy and Biochemistry, Johannes Gutenberg-University Mainz, 55128 Mainz, Germany; 4 W. Harry Feinstone Department of Molecular Microbiology and Immunology, Bloomberg School of Public Health, Johns Hopkins University, 21205 Baltimore, MD, USA

## Abstract

*Aedes aegypti* mosquitoes are important vectors of viral diseases. Mosquito host factors play key roles in virus control and it has been suggested that dengue virus replication is regulated by Dnmt2-mediated DNA methylation. However, recent studies have shown that Dnmt2 is a tRNA methyltransferase and that Dnmt2-dependent methylomes lack defined DNA methylation patterns, thus necessitating a systematic re-evaluation of the mosquito genome methylation status. We have now searched the *Ae. aegypti* genome for candidate DNA modification enzymes. This failed to reveal any known (cytosine-5) DNA methyltransferases, but identified homologues for the Dnmt2 tRNA methyltransferase, the Mettl4 (adenine-6) DNA methyltransferase, and the Tet DNA demethylase. All genes were expressed at variable levels throughout mosquito development. Mass spectrometry demonstrated that DNA methylation levels were several orders of magnitude below the levels that are usually detected in organisms with DNA methylation-dependent epigenetic regulation. Furthermore, whole-genome bisulfite sequencing failed to reveal any evidence of defined DNA methylation patterns. These results suggest that the *Ae. aegypti* genome is unmethylated. Interestingly, additional RNA bisulfite sequencing provided first evidence for Dnmt2-mediated tRNA methylation in mosquitoes. These findings have important implications for understanding the mechanism of Dnmt2-dependent virus regulation.

*Aedes aegypti* mosquitoes are important vectors of viral diseases, such as yellow fever, dengue, chikungunya and Zika, which have a significant impact on human morbidity and mortality^1^. The incidence of dengue has grown around the world in the last decades, and since 2015, an outbreak of Zika virus infections in Brazil, with increasing number of microcephaly-associated cases, has raised serious worldwide public health concerns^2^. Vector control strategies, such as the use of insecticide applications have proven ineffective due to the rapid development of resistance by the mosquitoes^3^. Thus, it has become mandatory that we better understand the biology of the vector/pathogen interactions.

The viruses display the remarkable capacity to survive and replicate in two very different host organisms, which is accomplished by a genome encoding a mere 10 proteins^4^. To be successful, the viruses must engage in molecular interactions that allow the virus to utilize existing host cellular systems and factors to perpetuate the virus lifecycle. It has become clear that mosquito host factors are involved in the RNA replication, transcription and translation of several flaviviruses^5^. In this regard, dengue virus (DENV) replication has been recently shown to be influenced by AaDnmt2 and it was suggested that this could be mediated by Dnmt2-dependent DNA methylation^6^. However, the DNA methylation status of the *Ae. aegypti* genome has so far only been investigated by indirect methods^7^.

DNA methylation is a conserved epigenetic modification with complex regulatory functions^8^,^9^,^10^. Most methylation marks on eukaryotic genomes are found at cytosine-5 (m5C) and are catalyzed by the Dnmt1 and Dnmt3 DNA methyltransferases^11^. In addition, Dnmt2 contains all the signature motifs of a DNA methyltransferase, but the enzyme actually functions as a (cytosine-5) tRNA methyltransferase^11^,^12^,^13^. More recently, very low levels of adenine-6 DNA methylation (m6A) have also been described in various eukaryotic genomes^14^. This modification is catalyzed by a different family of enzymes and may contribute to epigenetic gene regulation^15^.

Dnmt2 as a widely conserved enzyme and its potential DNA methyltransferase activity has been discussed controversially over a considerable period of time^12^. The functional characterization of the enzyme was greatly aided by the availability of Dnmt2-deficient *Drosophila* strains. Detailed molecular analyses have shown that *Drosophila* Dnmt2 is not a DNA methyltransferase, but a C38 tRNA methyltransferase^16^,^17^,^18^. Flies lacking Dnmt2 are viable and fertile but have shown subtle phenotypic effects in a variety of assays^16^,^17^. Loss of Dnmt2 promotes endonucleolytic cleavage of tRNA fragments in flies^17^, and results in the loss of RNA-dependent gene regulation^19^. While Dnmt2 has also been shown to contribute to RNA virus control in *Drosophila*, the underlying mechanisms are fundamentally different from classical epigenetic regulation by DNA modifications^20^. These findings have prompted us to directly investigate the DNA methylation status of the *Ae. aegypti* genome.

## Results

### Conservation of candidate DNA modification enzymes in *Ae. aegypti*

**Figure 1:**
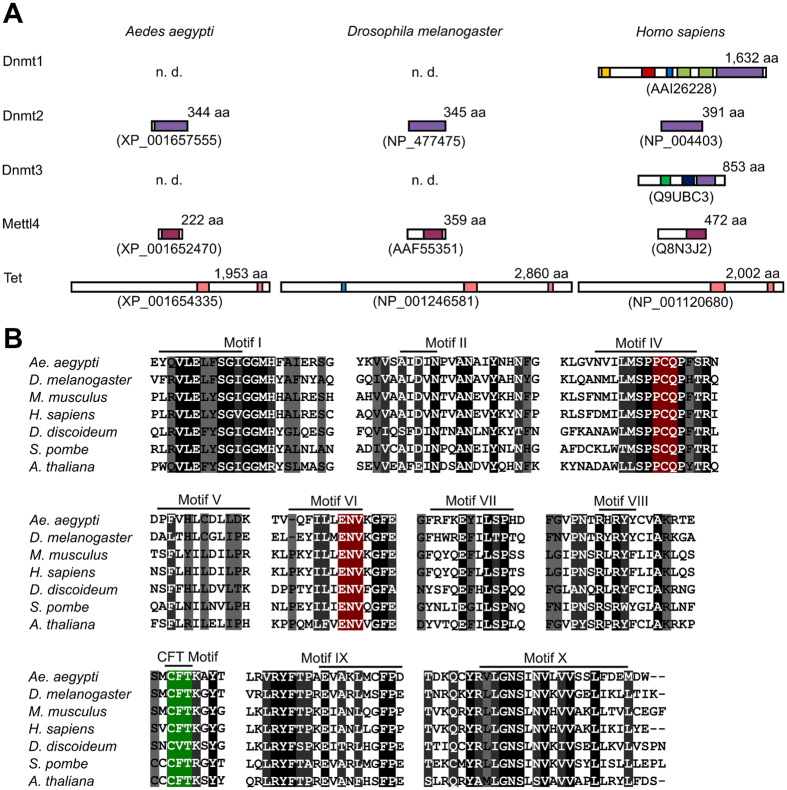
Conservation of Dnmt2, Mettl4 and Tet in *Ae. aegypti*. (**A**) An analysis of the *Ae. aegypti* peptide database version 3.3 revealed the presence of single homologues for Dnmt2, Mettl4 and Tet, respectively. Conserved domains are shown as colored boxes. Dnmt1: orange - DMAP1 binding domain, red - replication foci domain, blue - CXXC zinc finger domain, green - bromo adjacent homology domain, purple - catalytic domain. Dnmt2: purple - catalytic domain. Dnmt3: green - PWWP domain, blue - zinc finger domain, purple - catalytic domain. Mettl4: magenta - catalytic domain. Tet: blue - CXXC zinc finger domain, pink-catalytic domain. Accession numbers are provided below the protein symbols. n.d., not detected. (**B**) Multiple sequence alignment of the DNA methyltransferase motifs of AaDnmt2 and the corresponding sequences of several Dnmt2 homologs with experimentally validated C38 tRNA methyltransferase activity. Black background denotes 100% identity, dark grey background 80–99% identity and grey background 60–79% identity. The catalytic motifs are highlighted in red, the Dnmt2-specific CFT motif^34^ is highlighted in green. For additional details, see Fig. S1.

Previous studies have suggested that the *Ae. aegypti* genome is methylated and that Dnmt2-mediated methylation is involved in DENV replication in the mosquito vector^6^,^7^. However, important mechanistic details remained to be elucidated. We therefore performed a systematic analysis of the mosquito genome for candidate genes that are known to be involved in DNA modification. This approach failed to identify homologues of the known (cytosine-5) DNA methyltransferases, Dnmt1 and Dnmt3 (Fig. 1A). We could, however, detect a highly conserved Dnmt2 homologue (Fig. 1A), which contains all the conserved motifs of Dnmt2 enzymes with experimentally confirmed C38 tRNA methyltransferase activity (Fig. 1B, Fig. S1). Finally, we also detected homologues of Mettl4 and Tet in the *Ae. aegypti* genome (Fig. 1A). These genes have been implicated in (adenine-6) DNA methylation and demethylation, respectively^14^. (Adenine-6) methylation has been shown to be present in the *Drosophila* genome, but appears to be restricted to a small window of time during early embryonic development^21^.

### Expression of AaDnmt2, AaMettl4 and AaTet in *Ae. aegypti*

**Figure 2:**
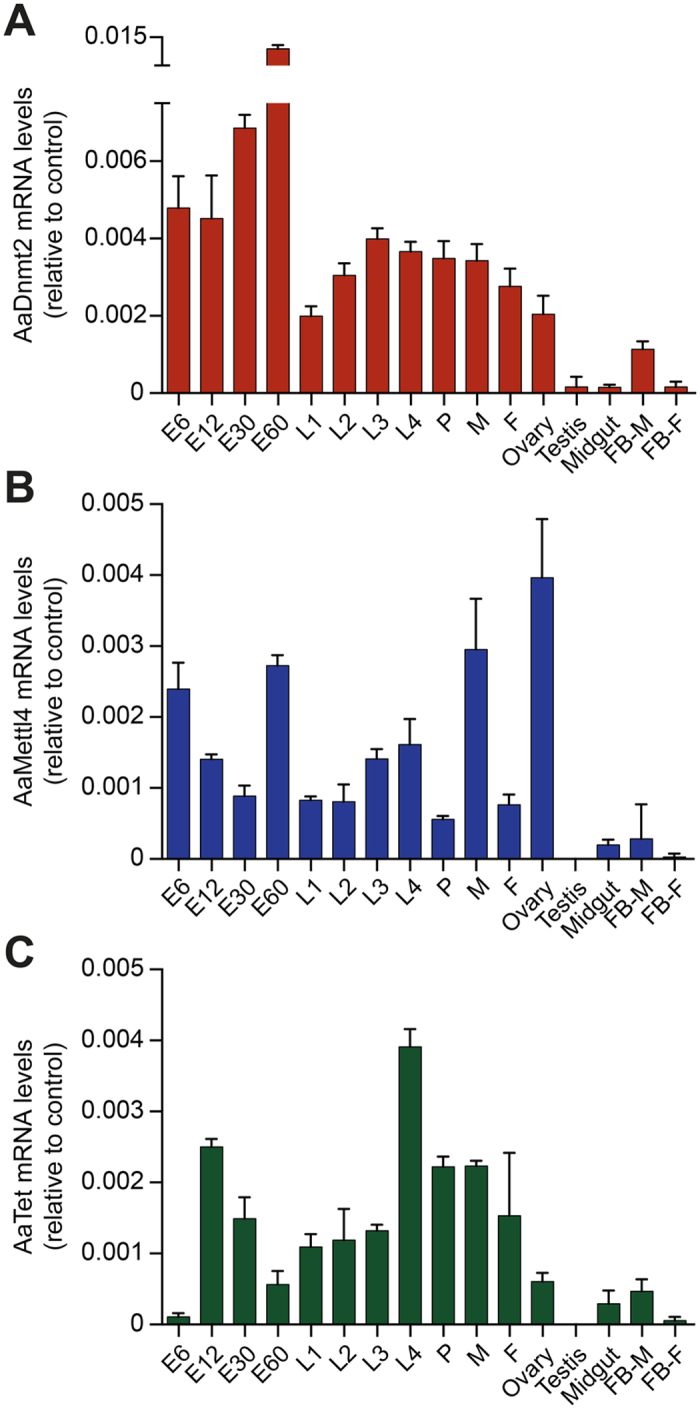
Expression of candidate DNA modification enzymes during different mosquito life stages and adult tissues. mRNA expression levels were determined by qRT-PCR for (**A**) AaDNMT2, (**B**) AaMettl4 and (**C**) AaTet using AaRP-49 mRNA for normalization. Bars represent relative expression levels with standard errors. Stages of embryo development are indicated in hours after oviposition. 1^st^ instar larvae (L1), 2^nd^ instar larvae (L2), 3^rd^ instar larvae (L3), 4^th^ instar larvae (L4), pupae (P), male (M), female (F), fat body (FB). Values are means of triplicate samples.

AaDnmt2 has been shown to be expressed in larval and adult stages of *Ae. aegypti* development, with highest levels in ovaries^6^. However, the expression of AaMettl4 and AaTet has not been investigated yet. We therefore performed quantitative real-time to evaluate the mRNA levels of all three genes during various stages of development and in various tissues. The expression levels of AaDnmt2 in all four embryonic stages were higher than in the four larval stages, pupae, and male or female adults, and reached their peak in 60 h embryos (Fig. 2A). Importantly, when we analyzed the expression of AaDnmt2 in individual dissected organs, moderate levels of mRNA were detected in the ovary and male fat bodies, whereas mRNA levels were low in testis, midgut and female fat bodies (Fig. 2A). Similar mRNA expression profiles were observed for AaMettl4 and AaTet (Fig. 2B,C), except for AaTet in 6 h embryos, where only very low levels were detected (Fig. 2B). The expression of AaMettl4 and AaTet in most of the adult tissues followed the pattern of AaDnmt2 expression. However, the mRNA levels of AaMettl4 in the ovary were significantly increased (Fig. 2C). Together, these data provide a detailed characterization of the developmental expression pattern for all three enzymes.

### Mass spectrometry analysis of DNA methylation

**Figure 3:**
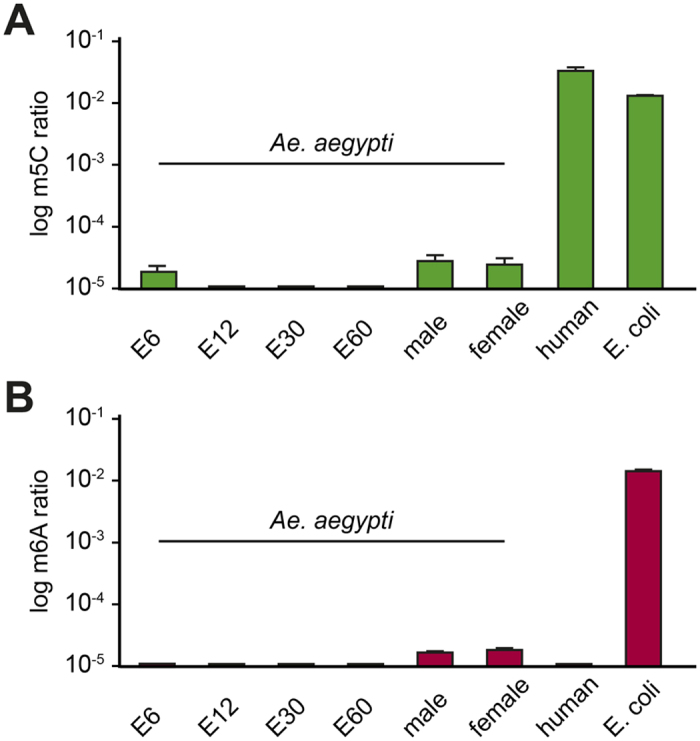
DNA methylation analysis of *Ae. aegypti* genomic DNA. Quantitative analysis of (**A**) 5-methylcytosine and (**B**) 6-methyladenine levels by mass spectrometry. Samples were from various developmental stages and tissues, as indicated. All results represent average values from three or more measurements. Standard deviations are indicated by error bars.

In order to investigate the functionality of the *Ae. aegypti* DNA methylation system, we used highly sensitive mass spectrometry to quantitatively determine DNA methylation levels during various stages of embryonic development and in whole adult animals. In addition, we included human blood DNA as a positive control. The results showed robust (5.3%) levels of cytosine methylation for human blood, but showed only marginal levels (<50 ppm) of this modification for the various *Ae. aegypti* samples (Fig. 3A). We also used mass spectrometry to quantitatively determine adenine methylation levels in the same samples. For this analysis, we included *E. coli* DNA as a positive control, as adenine methylation is known to be prevalent in bacterial DNA^22^. Indeed, our results showed robust (1.7%) levels of adenine methylation for the *E. coli* sample, but only marginal levels (<50 ppm) for the *Ae. aegypti* samples (Fig. 3B). The somewhat elevated methylation levels that were consistently observed in adults are likely caused by bacterial DNA from the mosquito microbiota. Together, our results suggest that the mosquito genome is either methylated at extremely low levels or not at all.

### Sequencing-based analysis of cytosine-5 DNA methylation patterns

To further investigate cytosine methylation in the *Ae. aegypti* genome, we used whole-genome bisulfite sequencing. This method represents the current gold standard for DNA methylation analysis and allows the generation of genome-wide methylation maps at single-base resolution^23^. Importantly, because DNA methylation is detected in its species-specific DNA sequence context, this method also precludes contaminations from bacterial DNA in the methylation analysis.

DNA for whole-genome bisulfite sequencing was prepared from mixed (1:1) adult males and females. For controls, 1% of unmethylated bacteriophage lambda DNA (negative control) and 8% of human blood DNA (positive control) were spiked into the *Ae. aegypti* sample prior to bisulfite conversion. Sequencing on an Illumina X-Ten platform generated 494 million read pairs that were subsequently mapped to the *Ae. aegypti*, lambda and human reference genomes, resulting in average CpG coverages of 26× (*Ae. aegypti*), 3670× (lambda) and 1× (human), respectively.

**Figure 4:**
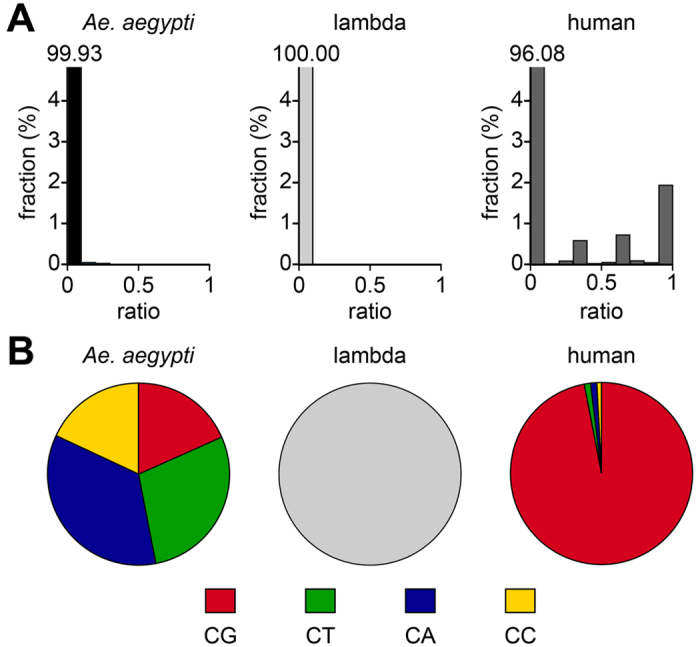
Characterization of the *Ae. aegypti* methylome by whole-genome bisulfite sequencing. (**A**) Average methylation levels were determined for all cytosine residues and then distributed into bins with increasing methylation ratios. For comparison, the corresponding data is also shown for bacteriophage lambda (negative control) and human blood (positive control) DNA that was spiked into the *Ae. aegypti* sample prior to bisulfite conversion. The actual numerical values of the first bins are indicated above the corresponding bars. (**B**) Dinucleotide sequence context of unconverted cytosines. No unconverted cytosines were found in the lambda dataset.

A detailed analysis of the *Ae. aegypti* data showed that the vast majority (99.9%) of cytosine residues appeared completely unmethylated (ratio <0.1), while only 0.0005% showed a non-conversion ratio >0.5 (Fig. 4A). This distribution was similar to the spiked-in negative control (Fig. 4A), but substantially different from the positive control, which showed complete methylation (ratio >0.9) for 2.0% of the cytosine residues (Fig. 4A). Pronounced differences between the mosquito and human samples were also detectable for the dinucleotide sequence context of non-converted cytosine residues. While the human blood data showed the known enrichment for CpG dinucleotides, no enrichment could be detected in the *Ae. aegypti* and lambda (negative control) datasets (Fig. 4B). Together, these results strongly suggest that the mosquito genome is unmethylated at cytosine residues. As such, AaDnmt2-dependent regulation of Dengue virus replication^6^ would have to be independent of cytosine DNA methylation.

### Sequencing-based analysis of tRNA methylation patterns

**Figure 5:**
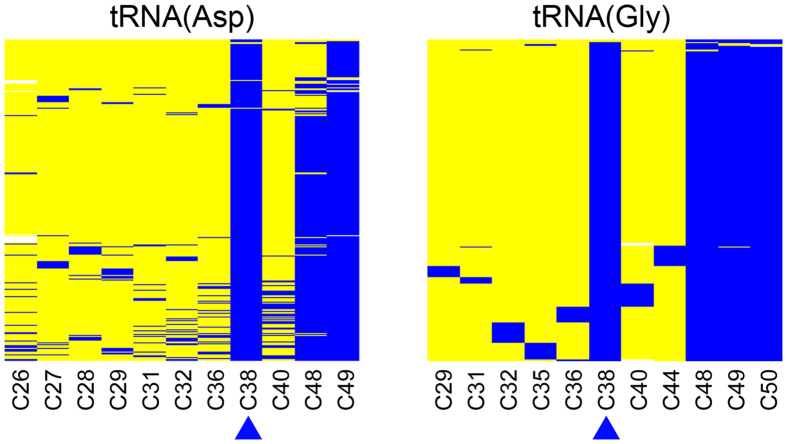
Methylation analysis of tRNA(Asp) and tRNA(Gly) by deep RNA bisulfite sequencing. Each row represents a sequence read, each column a cytosine residue. Yellow boxes represent unmethylated cytosine residues, blue boxes indicate methylated cytosine residues, sequencing gaps are shown in white. The positional number of each specific cytosine residue is shown at the bottom and the Dnmt2 target site (C38) is highlighted by a blue arrowhead. Sequencing coverages were 713× for tRNA(Asp) and 462× for tRNA(Gly).

Research over the past few years strongly suggested that Dnmt2 is a tRNA methyltransferase, rather than a DNA methyltransferase^16^,^18^. Moreover, our previous work in *Drosophila* has shown that Dnmt2 specifically methylates C38 of tRNA(Asp), tRNA(Gly) and tRNA(Val)^17^. As Dnmt2-dependent C38 methylation is widely conserved in evolution (Fig. 1B), it is reasonable to assume that it was also present in *Ae. aegypti*. We therefore established RNA bisulfite sequencing assays to investigate the methylation patterns of known Dnmt2 substrate tRNAs in the mosquito. Deep sequencing of tRNA(Asp) and tRNA(Gly) amplicons demonstrated the presence of conserved tRNA methylation patterns in adult mosquitoes (Fig. 5). This included almost complete methylation of C38 (Fig. 5), thus demonstrating the conservation of this modification in *Ae. aegypti.* These findings have important implications for understanding the mechanism of AaDnmt2-dependent virus control, as discussed below.

## Discussion

(Cytosine-5) DNA methylation is an important epigenetic modification with regulatory functions in many biological processes, including cellular differentiation, X-chromosome inactivation and transposon control^8^,^9^,^10^. Interestingly, recent results also suggest epigenetic regulatory functions for (adenine-6) DNA methylation^14^. Despite the considerable evolutionary conservation of both cytosine and adenine methylation, it is important to notice that these modifications do not have a conserved essential function, as several eukaryotic organisms are known to have unmethylated genomes^18^.

Our data suggest that the *Ae. aegypti* genome is largely unmethylated. Very low levels of 6 mA are consistent with findings in *Drosophila*, where adenine methylation could only be detected by highly sensitive methods and during very early stages of development^21^. While m6A has been implicated in transposon regulation in *Drosophila*, the modification showed a positive correlation with transposon expression, which is currently not understood mechanistically^21^. Similarly, our results obtained for 5 mC with *Ae. aegypti* DNA were consistent with our previous findings in *Drosophila*^18^ and suggest that the mosquito genome is not methylated at cytosine residues. The extremely low methylation levels detected in our analysis are unlikely to be of biological relevance and may have been caused by spurious enzyme activity and/or by contamination with highly modified bacterial DNA.

The absence of detectable DNA methylation in *Ae. aegypti* requires a novel mechanistic explanation for the observed role of AaDnmt2 in DENV replication^6^. This could be provided by Dnmt2-mediated tRNA methylation. Indeed, we could show that C38 methylation of tRNA(Asp) and tRNA(Gly) is conserved in *Ae. aegypti.* Our attempts to directly demonstrate the dependency of C38 methylation on AaDnmt2 by RNAi were unsuccessful (data not shown). Also, specific inhibitors for the establishment of AaDnmt2 function are not available, as azacytidine has an indirect mode of action with complex effects on many cellular pathways^24^,^25^. However, the AaDnmt2 signature motifs are highly conserved with Dnmt2 homologs that have experimentally been validated as C38 tRNA methyltransferases (Fig. 1B), which strongly suggests that C38 tRNA methylation in *Ae. aegypti* is catalyzed by AaDnmt2. A more detailed functional characterization of AaDnmt2 will require the generation of genetically mutant alleles.

In this context it is particularly interesting to note that Dnmt2-deficient *Drosophila* have shown a pronounced defect in their ability to control the proliferation of RNA viruses^20^. More specifically, it was demonstrated that Dnmt2 mutant flies accumulate high levels of *Drosophila* C virus (DCV) and that Dnmt2 binds to DCV RNAs^20^. While our results indicate only relatively low levels of AaDnmt2 mRNA in the midgut and female fat body of uninfected mosquitoes, expression became moderately, but significantly induced in midguts from DENV-infected mosquitoes (Fig. S2). As DENV replication requires many RNA-RNA cis interactions of the DENV RNA^26^, it is conceivable that AaDnmt2 could either facilitate or disturb some of these interactions.

## Methods

### Mosquito culture and sample collection

*Ae. aegypti* (Red Eye strain) were reared in an insectary at the Federal University of Rio de Janeiro, Brazil, at 28  ±  2 °C and 80  ±  5% humidity on a 12 h light-dark cycle. The larvae were fed with dog chow. The different embryo development stages were collected as previously reported^27^ and optical images were acquired in a Leica stereomicroscope M205 (Wetzlar, Germany). Mosquito life stages larva 1 (L1), larva 2 (L2), larva 3 (L3), larva 4 (L4) and pupa (P) were collected according to standard protocols and macroscopic observation. Three- to four-day-old male and female mosquitoes were used in the experiments. The mosquitoes were cold-anesthetized in phosphate buffered saline to dissect the ovary, testis, midgut and fat body.

### Gene expression analysis

Total RNA from all biological samples were extracted with TRIZOL reagent (Invitrogen, California, USA) following the manufacturer’s protocol. RNA was treated with DNase I (Qiagen, Hilden, Germany) and the first-strand cDNA synthesis was carried out using First-strand cDNA Sythesis Kit (Invitrogen, California, USA). The amplification efficiency of each gene was evaluated with serial dilution of cDNA and selected when efficiency was greater than 90%. Quantitative PCR was performed in a StepOnePlus Real Time PCR System (Applied Biosystems, St. Louis, MO) using Power SYBR Green PCR Master Mix (Applied Biosystems, St. Louis, MO) and 300 nM of forward and reverse primers with standard reaction conditions (20 seconds at 95 °C followed by 40 cycles of 95 °C for 1 second and 20 seconds at 60 °C followed by a melting curve). The comparative ΔΔCt method was used to evaluate changes in gene expression. The *Ae. aegypti* ribosomal protein 49 gene, RP-49, was used as endogenous control (accession number AAT45939). Each figure represents at least three biological replicates with three technical replicates. Primer sequences are provided in Supplementary Table 1.

### Measurement of global DNA methylation levels by mass spectrometry

Sample preparation and LC-MS/MS analyses were conducted as described previously^28^,^29^ using 50 ng/injection of an internal standard derived from ^13^C labeled total DNA of *Saccharomyces cerevisiae* and were performed on an Agilent 1260 LC system connected to an Agilent 6460 TripleQuad mass spectrometer (Agilent, Böblingen, Germany). N^6^-methyldeoxyadenosine (m^6^dA) content in the *S. cerevisiae* DNA digest was calibrated by a nucleoside standard (Sigma Aldrich, Steinheim, Germany). Quantification of 5-methyldeoxycytidine (m^5^dC) was achieved *via* a chemically synthesized triple deuterated internal standard as described recently^30^,^31^.

### Whole-genome bisulfite sequencing

Genomic DNA was purified from whole adult mosquitoes (equal numbers of males and females) using the DNeasy Blood & Tissue Kit (Qiagen) following the manufacturer’s instructions. As a spike-in control, unmethylated bacteriophage lambda DNA (Promega) and genomic DNA extracted from human blood (Blood & Cell Culture DNA Kit, Qiagen) were used. Library preparation was performed as described previously^18^. Paired-end sequencing was performed on an Illumina X-Ten system. Unique reads were trimmed to a maximum length of 80 bp and stretches of bases having a quality score <30 at read ends were removed. Reads were mapped using BSMAP 2.0^32^. Only reads mapping uniquely and with both read pairs having the correct distance were used in further analyses. Methylation rates were determined using a Python script distributed with the BSMAP package. To reduce the effects of sequencing errors, methylated Cs were only called when covered by >3 reads. The *Ae. aegypti* Liverpool genome sequence (AaegL3) was used as a reference.

### tRNA bisulfite sequencing

Whole adult mosquitoes (equal numbers of males and females) were mechanically homogenized in TRIzol (Invitrogen) using a pellet pestle. Total RNA was extracted following the manufacturer’s instructions. Bisulfite conversion was performed using the EZ RNA Methylation Kit (Zymo Research). Amplicons for 454 (Roche) sequencing were generated and analyzed as described previously^33^. Primer sequences are provided in Supplementary Table 1.

## Additional Information

**How to cite this article**: Falckenhayn, C. *et al*. Comprehensive DNA methylation analysis of the *Aedes aegypti* genome. *Sci. Rep.* **6**, 36444; doi: 10.1038/srep36444 (2016).

**Publisher’s note**: Springer Nature remains neutral with regard to jurisdictional claims in published maps and institutional affiliations.

## Supplementary Material

Supplementary Information

## References

[b1] Kraemer M. U. . The global distribution of the arbovirus vectors Aedes aegypti and Ae. albopictus. eLife 4, e08347 (2015). 26126267 10.7554/eLife.08347 PMC4493616

[b2] Ribeiro L. S., Marques R. E., Jesus A. M., Almeida R. P. & Teixeira M. M. Zika crisis in Brazil: challenges in research and development. Curr. Opin. Virol. 18, 76–81 (2016). 27179929 10.1016/j.coviro.2016.04.002

[b3] Owusu H. F., Jancaryova D., Malone D. & Muller P. Comparability between insecticide resistance bioassays for mosquito vectors: time to review current methodology? Parasit. Vectors 8, 357 (2015). 26148484 10.1186/s13071-015-0971-6 PMC4492098

[b4] Gebhard L. G., Filomatori C. V. & Gamarnik A. V. Functional RNA elements in the dengue virus genome. Viruses 3, 1739–1756 (2011). 21994804 10.3390/v3091739 PMC3187688

[b5] Doolittle J. M. & Gomez S. M. Mapping protein interactions between Dengue virus and its human and insect hosts. PLoS Negl. Trop. Dis. 5, e954 (2011). 21358811 10.1371/journal.pntd.0000954 PMC3039688

[b6] Zhang G., Hussain M., O’Neill S. L. & Asgari S. Wolbachia uses a host microRNA to regulate transcripts of a methyltransferase, contributing to dengue virus inhibition in Aedes aegypti. Proc. Natl. Acad. Sci. USA 110, 10276–10281 (2013). 23733960 10.1073/pnas.1303603110 PMC3690878

[b7] Ye Y. H. . Infection with a Virulent Strain of Disrupts Genome Wide-Patterns of Cytosine Methylation in the Mosquito. PLoS ONE 8, e66482 (2013). 23840485 10.1371/journal.pone.0066482 PMC3686743

[b8] Law J. A. & Jacobsen S. E. Establishing, maintaining and modifying DNA methylation patterns in plants and animals. Nat. Rev. Genet. 11, 204–220 (2010). 20142834 10.1038/nrg2719 PMC3034103

[b9] Deaton A. M. & Bird A. CpG islands and the regulation of transcription. Genes Dev. 25, 1010–1022 (2011). 21576262 10.1101/gad.2037511 PMC3093116

[b10] Bergman Y. & Cedar H. DNA methylation dynamics in health and disease. Nat. Struct. Mol. Biol. 20, 274–281 (2013). 23463312 10.1038/nsmb.2518

[b11] Goll M. G. & Bestor T. H. Eukaryotic cytosine methyltransferases. Annu. Rev. Biochem. 74, 481–514 (2005). 15952895 10.1146/annurev.biochem.74.010904.153721

[b12] Schaefer M. & Lyko F. Solving the Dnmt2 enigma. Chromosoma 119, 35–40 (2010). 19730874 10.1007/s00412-009-0240-6

[b13] Jeltsch A. . Mechanism and biological role of Dnmt2 in nucleic acid methylation. RNA Biol. 27, 1–16 (2016). 10.1080/15476286.2016.1191737 PMC5699548 27232191

[b14] Breiling A. & Lyko F. Epigenetic regulatory functions of DNA modifications: 5-methylcytosine and beyond. Epigenetics Chromatin 8, 24 (2015). 26195987 10.1186/s13072-015-0016-6 PMC4507326

[b15] Wu T. P. . DNA methylation on N(6)-adenine in mammalian embryonic stem cells. Nature 532, 329–333 (2016). 27027282 10.1038/nature17640 PMC4977844

[b16] Goll M. G. . Methylation of tRNAAsp by the DNA methyltransferase homolog Dnmt2. Science 311, 395–398 (2006). 16424344 10.1126/science.1120976

[b17] Schaefer M. . RNA methylation by Dnmt2 protects transfer RNAs against stress-induced cleavage. Genes Dev. 24, 1590–1595 (2010). 20679393 10.1101/gad.586710 PMC2912555

[b18] Raddatz G. . Dnmt2-dependent methylomes lack defined DNA methylation patterns. Proc. Natl. Acad. Sci. USA 110, 8627–8631 (2013). 23641003 10.1073/pnas.1306723110 PMC3666705

[b19] Durdevic Z., Mobin M. B., Hanna K., Lyko F. & Schaefer M. The RNA Methyltransferase Dnmt2 Is Required for Efficient Dicer-2-Dependent siRNA Pathway Activity in Drosophila. Cell Rep. 12, 931–937 (2013). 10.1016/j.celrep.2013.07.046 24012760

[b20] Durdevic Z. . Efficient RNA virus control in Drosophila requires the RNA methyltransferase Dnmt2. EMBO Rep. 14, 269–275 (2013). 23370384 10.1038/embor.2013.3 PMC3589093

[b21] Zhang G. . N6-methyladenine DNA Modification in Drosophila. Cell 161, 893–906 (2015). 25936838 10.1016/j.cell.2015.04.018

[b22] Ratel D., Ravanat J. L., Berger F. & Wion D. N6-methyladenine: the other methylated base of DNA. Bioessays 28, 309–315 (2006). 16479578 10.1002/bies.20342 PMC2754416

[b23] Lister R. & Ecker J. R. Finding the fifth base: genome-wide sequencing of cytosine methylation. Genome Res. 19, 959–966 (2009). 19273618 10.1101/gr.083451.108 PMC3807530

[b24] Stresemann C. & Lyko F. Modes of action of the DNA methyltransferase inhibitors azacytidine and decitabine. Int. J. Cancer 123, 8–13 (2008). 18425818 10.1002/ijc.23607

[b25] Schaefer M., Hagemann S., Hanna K. & Lyko F. Azacytidine inhibits RNA methylation at DNMT2 target sites in human cancer cell lines. Cancer Res. 69, 8127–8132 (2009). 19808971 10.1158/0008-5472.CAN-09-0458

[b26] Alcaraz-Estrada S. L., Yocupicio-Monroy M. & del Angel R. M. Insights into dengue virus genome replication. Future Virol. 5, 575–592 (2010).

[b27] Vital W. . Germ band retraction as a landmark in glucose metabolism during Aedes aegypti embryogenesis. BMC Dev. Biol. 10, 25 (2010). 20184739 10.1186/1471-213X-10-25 PMC2838828

[b28] Kellner S. . Absolute and relative quantification of RNA modifications via biosynthetic isotopomers. Nucleic Acids Res. 42, e142 (2014). 25129236 10.1093/nar/gku733 PMC4191383

[b29] Vogt G. . The marbled crayfish as a paradigm for saltational speciation by autopolyploidy and parthenogenesis in animals. Biol. Open 4, 1583–1594 (2015). 26519519 10.1242/bio.014241 PMC4728364

[b30] Pfaffeneder T. . Tet oxidizes thymine to 5-hydroxymethyluracil in mouse embryonic stem cell DNA. Nat. Chem. Biol. 10, 574–581 (2014). 24838012 10.1038/nchembio.1532

[b31] Schmid K. . Variable presence of 5-methylcytosine in commercial RNA and DNA. RNA Biol. 12, 1152–1158 (2015). 26274337 10.1080/15476286.2015.1076612 PMC4829282

[b32] Xi Y. & Li W. BSMAP: whole genome bisulfite sequence MAPping program. BMC Bioinformatics 10, 232 (2009). 19635165 10.1186/1471-2105-10-232 PMC2724425

[b33] Tuorto F. . RNA cytosine methylation by Dnmt2 and NSun2 promotes tRNA stability and protein synthesis. Nat. Struct. Mol. Biol. 19, 900–905 (2012). 22885326 10.1038/nsmb.2357

[b34] Jurkowski T. P. & Jeltsch A. On the evolutionary origin of eukaryotic DNA methyltransferases and Dnmt2. PLoS ONE 6, e28104 (2011). 22140515 10.1371/journal.pone.0028104 PMC3227630

