# Supplementary material for: Comprehensive DNA methylation analysis of the Aedes aegypti genome
Source: Sci Rep. 2016 Nov 2;6:36444. doi: 10.1038/srep36444 (PMC5090363; doi:10.1038/srep36444)
Supplement: Supplementary Information [file srep36444-s1.pdf]

Supplementary information

Comprehensive DNA methylation analysis of the *Aedes aegypti* genome

Cassandra Falckenhayn, Vitor Coutinho Carneiro, Anderson de Mendonça Amarante, Katharina Schmid, Katharina Hanna, Seokyoung Kang, Mark Helm, George Dimopoulos, Marcelo Rosado Fantappié, and Frank Lyko

Contents:

Supplementary Figures 1 and 2

Supplementary Table 1

Supplementary references

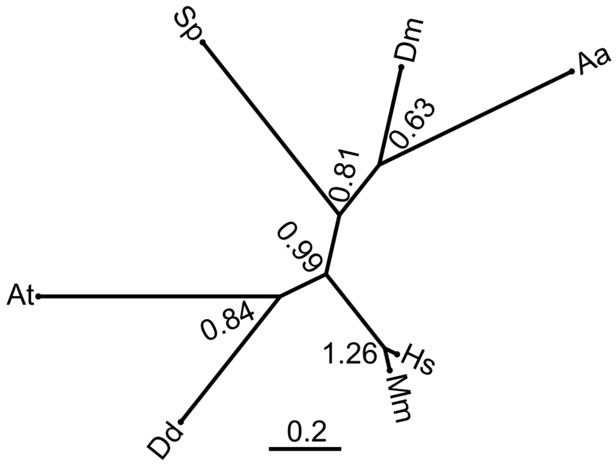

Figure S1. Phylogenetic tree of the Dnmt2 proteins from *Aedes aegypti* (Aa, XP_001657555), *Drosophila melanogaster* (Dm, NP_477475), *Homo sapiens* (Hs, NP_004403), *Mus musculus* (Mm, O55055), *Dictyostelium discoideum* (Dd, Q54JH6), *Arabidopsis thaliana* (At, OAO96001) and *Schizosaccharomyces pombe* (Sp, P40999). The multiple sequence alignment of the conserved amino acid sequence motifs was reduced to the homologous parts[1] and the phylogenetic tree was constructed by the Maximum-Likelihood method using PhyML 3.0 (ref. 2).

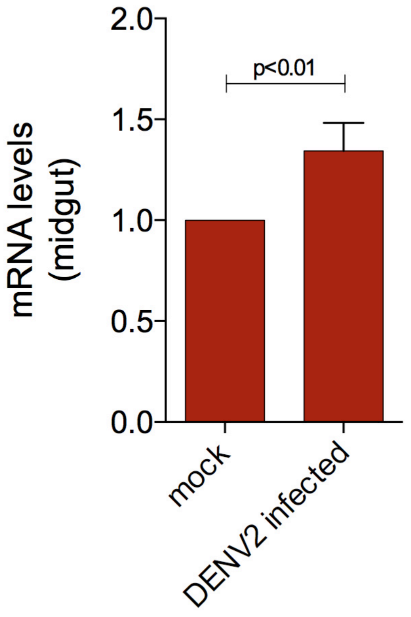

Figure S2. Induction of AaDnmt2 expression in midguts from DENV2-infected mosquitoes. mRNA expression levels were determined by qRT-PCR in multiple biological replicates from mock-infected and DENV2-infected mosquitoes. Virus infection was confirmed by plaque assays, statistical significance was determined by a Mann-Whitney test.

Supplementary Table 1. Primer sequences.

Primer	5'-3' sequence
Aedes_DNMT2_qRT-PCR_FW (863)	TGAGAAGTCTGAGAGTCCGATAC
Aedes_DNMT2_qRT-PCR_RV (993)	ATTGATGCTGTTGCCAAGAAC
Aedes_METTL4_qRT-PCR_FW (222)	CCAATTCGCCAAGTCACATTG
Aedes_METTL4_qRT-PCR_RV (325)	CTCATTGAAGGCACAAACCG
Aedes_TET_qRT-PCR_FW (2278)	CTTGGTCTCGGTGGCTATC
Aedes_TET_qRT-PCR_RV (2395)	TGATGTGGAAGGTGTGGATTG
AaRP49_qPCR_FW	GCTATGACAAGCTTGCCCCCA
AaRP49_qPCR_RV	TCATCAGCACCTCCAGCTC
Aedes_Asp_FW (454)	TGGTAGTATAGTGGTAAGTAT
Aedes_Asp_RV (454)	CTCCCCAACAAAAAATCA
Aedes_Gly_FW (454)	GTGGTTTAGTGGTAGAATGT
Aedes_Gly_RV (454)	TACATCAATCAAAAATCA

Supplementary references

1. Jurkowski, T. P. & Jeltsch, A. On the evolutionary origin of eukaryotic DNA methyltransferases and Dnmt2. *PLoS ONE* **6**, e28104 (2011).
2. Guindon, S. *et al.* New algorithms and methods to estimate maximum-likelihood phylogenies: assessing the performance of PhyML 3.0. *Syst. Biol.* **59**, 307-321 (2010).
